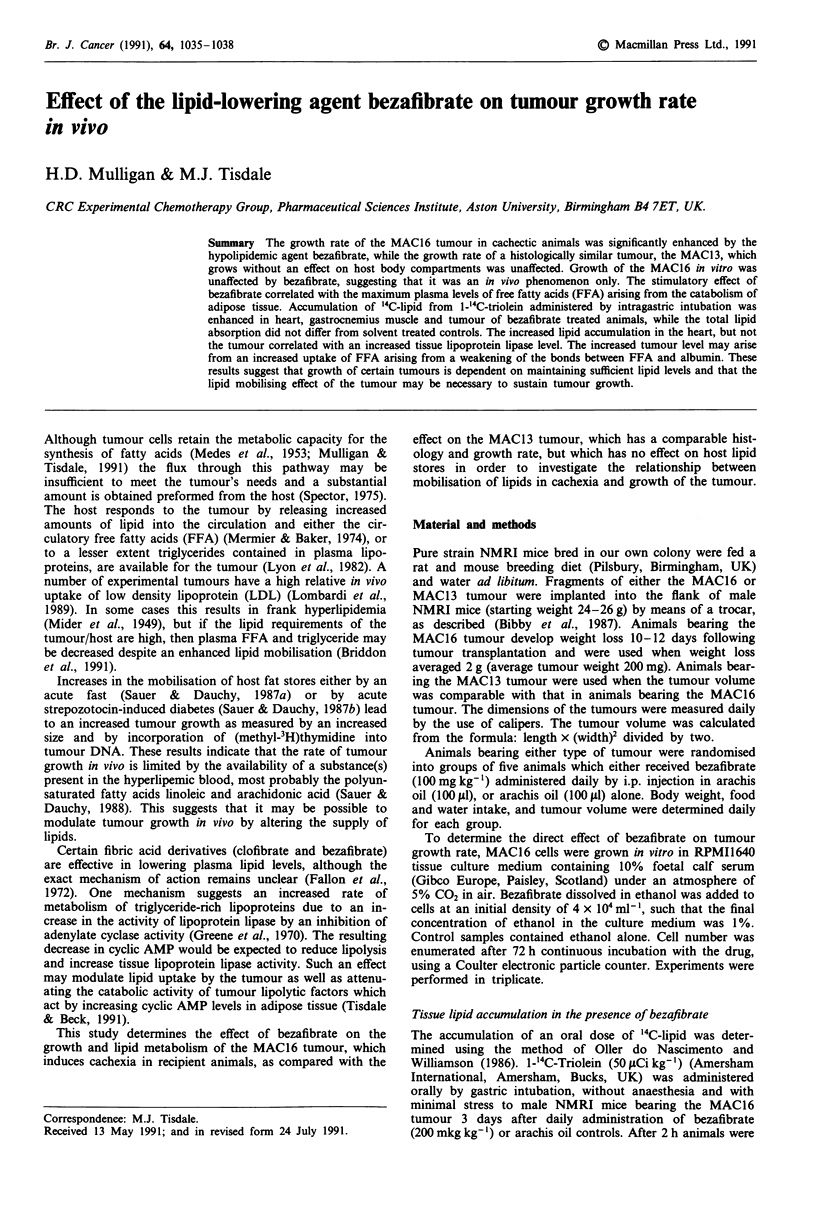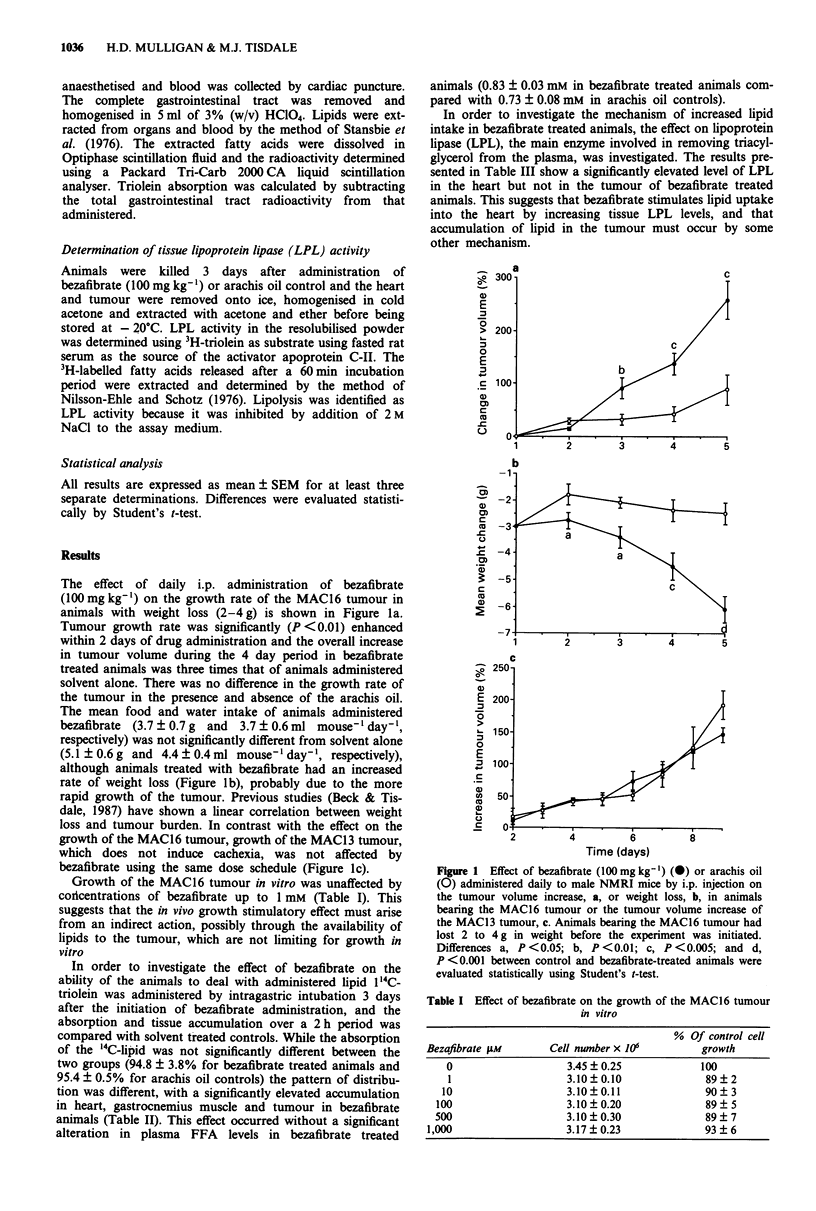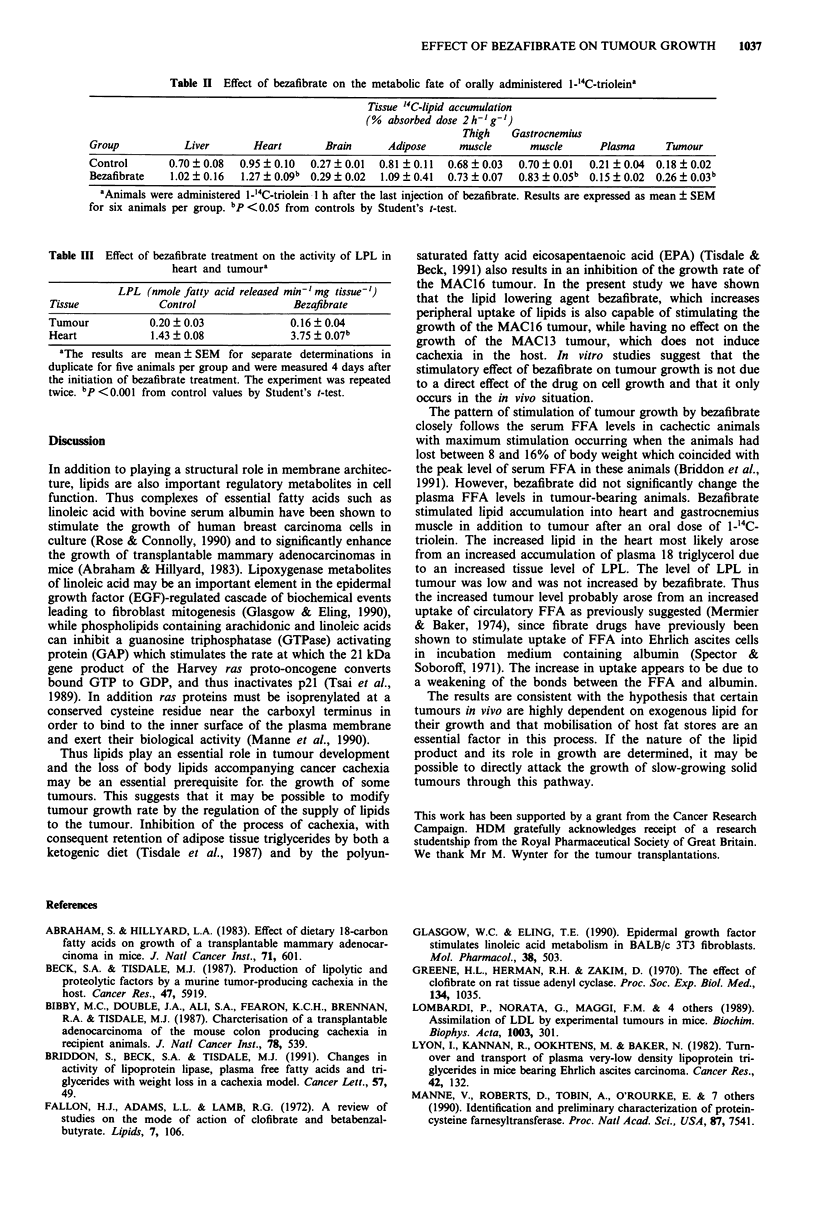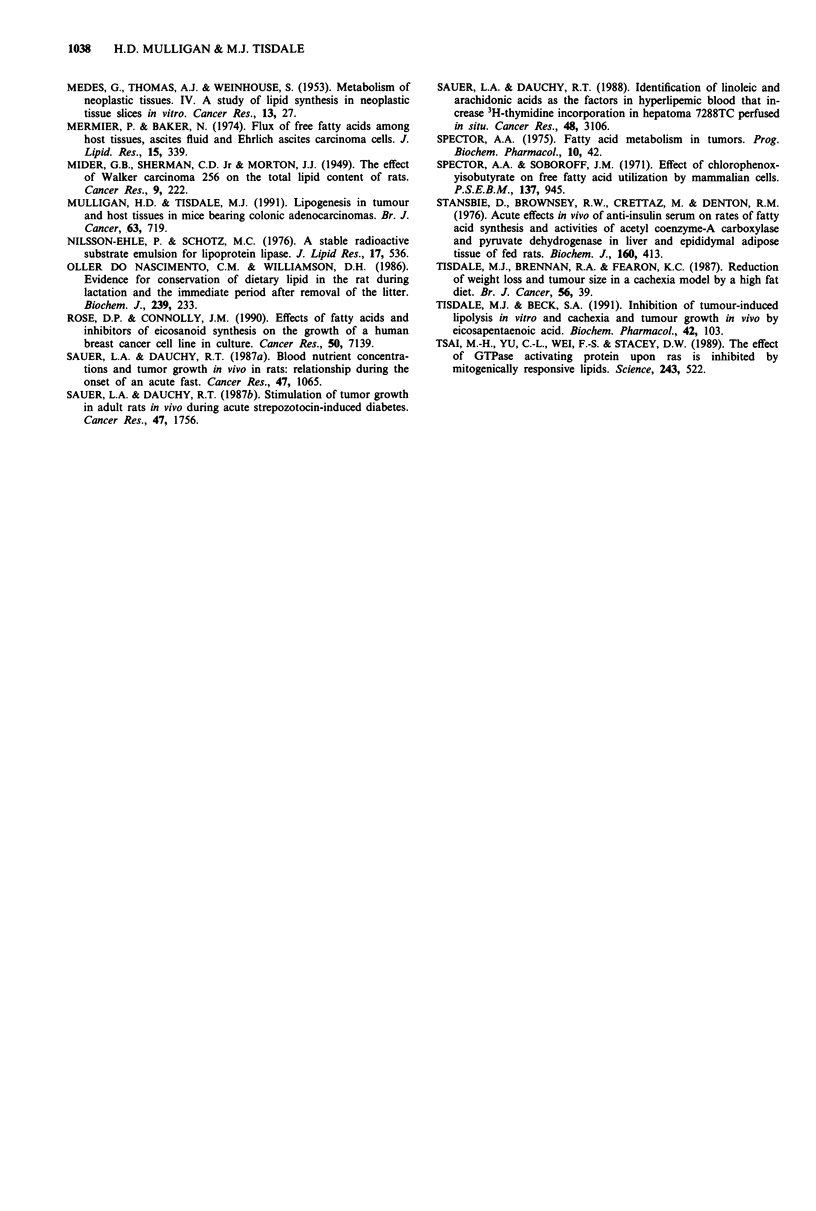# Effect of the lipid-lowering agent bezafibrate on tumour growth rate in vivo.

**DOI:** 10.1038/bjc.1991.460

**Published:** 1991-12

**Authors:** H. D. Mulligan, M. J. Tisdale

**Affiliations:** Pharmaceutical Sciences Institute, Aston University, Birmingham, UK.

## Abstract

The growth rate of the MAC16 tumour in cachectic animals was significantly enhanced by the hypolipidemic agent bezafibrate, while the growth rate of a histologically similar tumour, the MAC13, which grows without an effect on host body compartments was unaffected. Growth of the MAC16 in vitro was unaffected by bezafibrate, suggesting that it was an in vivo phenomenon only. The stimulatory effect of bezafibrate correlated with the maximum plasma levels of free fatty acids (FFA) arising from the catabolism of adipose tissue. Accumulation of 14C-lipid from 1-14C-triolein administered by intragastric intubation was enhanced in heart, gastrocnemius muscle and tumour of bezafibrate treated animals, while the total lipid absorption did not differ from solvent treated controls. The increased lipid accumulation in the heart, but not the tumour correlated with an increased tissue lipoprotein lipase level. The increased tumour level may arise from an increased uptake of FFA arising from a weakening of the bonds between FFA and albumin. These results suggest that growth of certain tumours is dependent on maintaining sufficient lipid levels and that the lipid mobilising effect of the tumour may be necessary to sustain tumour growth.


Br. J. Cancer (1991), 64, 1035-1038 © Macmillan Press Ltd., 1991

### Effect of the lipid-lowering agent bezafibrate on tumour growth rate *in vivo*

H.D. Mulligan & M.J. Tisdale

*CRC Experimental Chemotherapy Group, Pharmaceutical Sciences Institute, Aston University, Birmingham B4 7ET, UK.*

**Summary** The growth rate of the MAC16 tumour in cachectic animals was significantly enhanced by the hypolipidemic agent bezafibrate, while the growth rate of a histologically similar tumour, the MAC13, which grows without an effect on host body compartments was unaffected. Growth of the MAC16 *in vitro* was unaffected by bezafibrate, suggesting that it was an *in vivo* phenomenon only. The stimulatory effect of bezafibrate correlated with the maximum plasma levels of free fatty acids (FFA) arising from the catabolism of adipose tissue. Accumulation of $^{14}$C-lipid from 1-$^{14}$C-triolein administered by intragastric intubation was enhanced in heart, gastrocnemius muscle and tumour of bezafibrate treated animals, while the total lipid absorption did not differ from solvent treated controls. The increased lipid accumulation in the heart, but not the tumour correlated with an increased tissue lipoprotein lipase level. The increased tumour level may arise from an increased uptake of FFA arising from a weakening of the bonds between FFA and albumin. These results suggest that growth of certain tumours is dependent on maintaining sufficient lipid levels and that the lipid mobilising effect of the tumour may be necessary to sustain tumour growth.

Although tumour cells retain the metabolic capacity for the synthesis of fatty acids (Medes *et al.*, 1953; Mulligan & Tisdale, 1991) the flux through this pathway may be insufficient to meet the tumour's needs and a substantial amount is obtained preformed from the host (Spector, 1975). The host responds to the tumour by releasing increased amounts of lipid into the circulation and either the circulatory free fatty acids (FFA) (Mermier & Baker, 1974), or to a lesser extent triglycerides contained in plasma lipoproteins, are available for the tumour (Lyon *et al.*, 1982). A number of experimental tumours have a high relative *in vivo* uptake of low density lipoprotein (LDL) (Lombardi *et al.*, 1989). In some cases this results in frank hyperlipidemia (Mider *et al.*, 1949), but if the lipid requirements of the tumour/host are high, then plasma FFA and triglyceride may be decreased despite an enhanced lipid mobilisation (Briddon *et al.*, 1991).

Increases in the mobilisation of host fat stores either by an acute fast (Sauer & Dauchy, 1987*a*) or by acute strepozotocin-induced diabetes (Sauer & Dauchy, 1987*b*) lead to an increased tumour growth as measured by an increased size and by incorporation of (methyl-$^3$H)thymidine into tumour DNA. These results indicate that the rate of tumour growth *in vivo* is limited by the availability of a substance(s) present in the hyperlipemic blood, most probably the polyunsaturated fatty acids linoleic and arachidonic acid (Sauer & Dauchy, 1988). This suggests that it may be possible to modulate tumour growth *in vivo* by altering the supply of lipids.

Certain fibric acid derivatives (clofibrate and bezafibrate) are effective in lowering plasma lipid levels, although the exact mechanism of action remains unclear (Fallon *et al.*, 1972). One mechanism suggests an increased rate of metabolism of triglyceride-rich lipoproteins due to an increase in the activity of lipoprotein lipase by an inhibition of adenylate cyclase activity (Greene *et al.*, 1970). The resulting decrease in cyclic AMP would be expected to reduce lipolysis and increase tissue lipoprotein lipase activity. Such an effect may modulate lipid uptake by the tumour as well as attenuating the catabolic activity of tumour lipolytic factors which act by increasing cyclic AMP levels in adipose tissue (Tisdale & Beck, 1991).

This study determines the effect of bezafibrate on the growth and lipid metabolism of the MAC16 tumour, which induces cachexia in recipient animals, as compared with the effect on the MAC13 tumour, which has a comparable histology and growth rate, but which has no effect on host lipid stores in order to investigate the relationship between mobilisation of lipids in cachexia and growth of the tumour.

Correspondence: M.J. Tisdale.
Received 13 May 1991; and in revised form 24 July 1991.

### Material and methods

Pure strain NMRI mice bred in our own colony were fed a rat and mouse breeding diet (Pilsbury, Birmingham, UK) and water *ad libitum*. Fragments of either the MAC16 or MAC13 tumour were implanted into the flank of male NMRI mice (starting weight 24–26 g) by means of a trocar, as described (Bibby *et al.*, 1987). Animals bearing the MAC16 tumour develop weight loss 10–12 days following tumour transplantation and were used when weight loss averaged 2 g (average tumour weight 200 mg). Animals bearing the MAC13 tumour were used when the tumour volume was comparable with that in animals bearing the MAC16 tumour. The dimensions of the tumours were measured daily by the use of calipers. The tumour volume was calculated from the formula: length × $(width)^2$ divided by two.

Animals bearing either type of tumour were randomised into groups of five animals which either received bezafibrate ($100 mg kg^{-1}$) administered daily by i.p. injection in arachis oil (100 µl), or arachis oil (100 µl) alone. Body weight, food and water intake, and tumour volume were determined daily for each group.

To determine the direct effect of bezafibrate on tumour growth rate, MAC16 cells were grown *in vitro* in RPMI1640 tissue culture medium containing 10% foetal calf serum (Gibco Europe, Paisley, Scotland) under an atmosphere of 5% $CO_2$ in air. Bezafibrate dissolved in ethanol was added to cells at an initial density of $4 \times 10^4 ml^{-1}$, such that the final concentration of ethanol in the culture medium was 1%. Control samples contained ethanol alone. Cell number was enumerated after 72 h continuous incubation with the drug, using a Coulter electronic particle counter. Experiments were performed in triplicate.

### *Tissue lipid accumulation in the presence of bezafibrate*

The accumulation of an oral dose of $^{14}$C-lipid was determined using the method of Oller do Nascimento and Williamson (1986). 1-$^{14}$C-Triolein ($50 \mu Ci kg^{-1}$) (Amersham International, Amersham, Bucks, UK) was administered orally by gastric intubation, without anaesthesia and with minimal stress to male NMRI mice bearing the MAC16 tumour 3 days after daily administration of bezafibrate ($200 mkg kg^{-1}$) or arachis oil controls. After 2 h animals were anaesthetised and blood was collected by cardiac puncture. The complete gastrointestinal tract was removed and homogenised in 5 ml of 3% (w/v) $HClO_4$. Lipids were extracted from organs and blood by the method of Stansbie *et al.* (1976). The extracted fatty acids were dissolved in Optiphase scintillation fluid and the radioactivity determined using a Packard Tri-Carb 2000 CA liquid scintillation analyser. Triolein absorption was calculated by subtracting the total gastrointestinal tract radioactivity from that administered.

*Determination of tissue lipoprotein lipase (LPL) activity*

Animals were killed 3 days after administration of bezafibrate (100 mg $kg^{-1}$) or arachis oil control and the heart and tumour were removed onto ice, homogenised in cold acetone and extracted with acetone and ether before being stored at − 20°C. LPL activity in the resolubilised powder was determined using $^{3}$H-triolein as substrate using fasted rat serum as the source of the activator apoprotein C-II. The $^{3}$H-labelled fatty acids released after a 60 min incubation period were extracted and determined by the method of Nilsson-Ehle and Schotz (1976). Lipolysis was identified as LPL activity because it was inhibited by addition of 2 M NaCl to the assay medium.

*Statistical analysis*

All results are expressed as mean ± SEM for at least three separate determinations. Differences were evaluated statistically by Student's *t*-test.

### Results

The effect of daily i.p. administration of bezafibrate (100 mg $kg^{-1}$) on the growth rate of the MAC16 tumour in animals with weight loss (2–4 g) is shown in Figure 1a. Tumour growth rate was significantly ($P < 0.01$) enhanced within 2 days of drug administration and the overall increase in tumour volume during the 4 day period in bezafibrate treated animals was three times that of animals administered solvent alone. There was no difference in the growth rate of the tumour in the presence and absence of the arachis oil. The mean food and water intake of animals administered bezafibrate (3.7 ± 0.7 g and 3.7 ± 0.6 ml $mouse^{-1}$ $day^{-1}$, respectively) was not significantly different from solvent alone (5.1 ± 0.6 g and 4.4 ± 0.4 ml $mouse^{-1}$ $day^{-1}$, respectively), although animals treated with bezafibrate had an increased rate of weight loss (Figure 1b), probably due to the more rapid growth of the tumour. Previous studies (Beck & Tisdale, 1987) have shown a linear correlation between weight loss and tumour burden. In contrast with the effect on the growth of the MAC16 tumour, growth of the MAC13 tumour, which does not induce cachexia, was not affected by bezafibrate using the same dose schedule (Figure 1c).

Growth of the MAC16 tumour *in vitro* was unaffected by concentrations of bezafibrate up to 1 mM (Table I). This suggests that the *in vivo* growth stimulatory effect must arise from an indirect action, possibly through the availability of lipids to the tumour, which are not limiting for growth *in vitro*

In order to investigate the effect of bezafibrate on the ability of the animals to deal with administered lipid 1$^{14}$C-triolein was administered by intragastric intubation 3 days after the initiation of bezafibrate administration, and the absorption and tissue accumulation over a 2 h period was compared with solvent treated controls. While the absorption of the $^{14}$C-lipid was not significantly different between the two groups (94.8 ± 3.8% for bezafibrate treated animals and 95.4 ± 0.5% for arachis oil controls) the pattern of distribution was different, with a significantly elevated accumulation in heart, gastrocnemius muscle and tumour in bezafibrate animals (Table II). This effect occurred without a significant alteration in plasma FFA levels in bezafibrate treated animals (0.83 ± 0.03 mM in bezafibrate treated animals compared with 0.73 ± 0.08 mM in arachis oil controls).

In order to investigate the mechanism of increased lipid intake in bezafibrate treated animals, the effect on lipoprotein lipase (LPL), the main enzyme involved in removing triacylglycerol from the plasma, was investigated. The results presented in Table III show a significantly elevated level of LPL in the heart but not in the tumour of bezafibrate treated animals. This suggests that bezafibrate stimulates lipid uptake into the heart by increasing tissue LPL levels, and that accumulation of lipid in the tumour must occur by some other mechanism.

**Figure 1** Effect of bezafibrate (100 mg $kg^{-1}$) (●) or arachis oil (○) administered daily to male NMRI mice by i.p. injection on the tumour volume increase, **a**, or weight loss, **b**, in animals bearing the MAC16 tumour or the tumour volume increase of the MAC13 tumour, **c**. Animals bearing the MAC16 tumour had lost 2 to 4 g in weight before the experiment was initiated. Differences a, $P < 0.05$; b, $P < 0.01$; c, $P < 0.005$; and d, $P < 0.001$ between control and bezafibrate-treated animals were evaluated statistically using Student's *t*-test.

**Table I** Effect of bezafibrate on the growth of the MAC16 tumour *in vitro*

| *Bezafibrate μM* | *Cell number × $10^6$* | *% Of control cell growth* |
|---|---|---|
| 0 | 3.45 ± 0.25 | 100 |
| 1 | 3.10 ± 0.10 | 89 ± 2 |
| 10 | 3.10 ± 0.11 | 90 ± 3 |
| 100 | 3.10 ± 0.20 | 89 ± 5 |
| 500 | 3.10 ± 0.30 | 89 ± 7 |
| 1,000 | 3.17 ± 0.23 | 93 ± 6 |

**Table II** Effect of bezafibrate on the metabolic fate of orally administered 1-$^{14}$C-triolein[a]

| | Tissue $^{14}$C-lipid accumulation (% absorbed dose 2 $h^{-1}$ $g^{-1}$) | | | | | | | |
|---|---|---|---|---|---|---|---|---|
| *Group* | *Liver* | *Heart* | *Brain* | *Adipose* | *Thigh muscle* | *Gastrocnemius muscle* | *Plasma* | *Tumour* |
| Control | 0.70 ± 0.08 | 0.95 ± 0.10 | 0.27 ± 0.01 | 0.81 ± 0.11 | 0.68 ± 0.03 | 0.70 ± 0.01 | 0.21 ± 0.04 | 0.18 ± 0.02 |
| Bezafibrate | 1.02 ± 0.16 | 1.27 ± 0.09[b] | 0.29 ± 0.02 | 1.09 ± 0.41 | 0.73 ± 0.07 | 0.83 ± 0.05[b] | 0.15 ± 0.02 | 0.26 ± 0.03[b] |

[a]Animals were administered 1-$^{14}$C-triolein 1 h after the last injection of bezafibrate. Results are expressed as mean ± SEM for six animals per group. [b]$P < 0.05$ from controls by Student's *t*-test.

**Table III** Effect of bezafibrate treatment on the activity of LPL in heart and tumour[a]

| | LPL (nmole fatty acid released $min^{-1}$ mg $tissue^{-1}$) | |
|---|---|---|
| *Tissue* | *Control* | *Bezafibrate* |
| Tumour | 0.20 ± 0.03 | 0.16 ± 0.04 |
| Heart | 1.43 ± 0.08 | 3.75 ± 0.07[b] |

[a]The results are mean ± SEM for separate determinations in duplicate for five animals per group and were measured 4 days after the initiation of bezafibrate treatment. The experiment was repeated twice. [b]$P < 0.001$ from control values by Student's *t*-test.

### Discussion

In addition to playing a structural role in membrane architecture, lipids are also important regulatory metabolites in cell function. Thus complexes of essential fatty acids such as linoleic acid with bovine serum albumin have been shown to stimulate the growth of human breast carcinoma cells in culture (Rose & Connolly, 1990) and to significantly enhance the growth of transplantable mammary adenocarcinomas in mice (Abraham & Hillyard, 1983). Lipoxygenase metabolites of linoleic acid may be an important element in the epidermal growth factor (EGF)-regulated cascade of biochemical events leading to fibroblast mitogenesis (Glasgow & Eling, 1990), while phospholipids containing arachidonic and linoleic acids can inhibit a guanosine triphosphatase (GTPase) activating protein (GAP) which stimulates the rate at which the 21 kDa gene product of the Harvey *ras* proto-oncogene converts bound GTP to GDP, and thus inactivates p21 (Tsai *et al.*, 1989). In addition *ras* proteins must be isoprenylated at a conserved cysteine residue near the carboxyl terminus in order to bind to the inner surface of the plasma membrane and exert their biological activity (Manne *et al.*, 1990).

Thus lipids play an essential role in tumour development and the loss of body lipids accompanying cancer cachexia may be an essential prerequisite for. the growth of some tumours. This suggests that it may be possible to modify tumour growth rate by the regulation of the supply of lipids to the tumour. Inhibition of the process of cachexia, with consequent retention of adipose tissue triglycerides by both a ketogenic diet (Tisdale *et al.*, 1987) and by the polyunsaturated fatty acid eicosapentaenoic acid (EPA) (Tisdale & Beck, 1991) also results in an inhibition of the growth rate of the MAC16 tumour. In the present study we have shown that the lipid lowering agent bezafibrate, which increases peripheral uptake of lipids is also capable of stimulating the growth of the MAC16 tumour, while having no effect on the growth of the MAC13 tumour, which does not induce cachexia in the host. *In vitro* studies suggest that the stimulatory effect of bezafibrate on tumour growth is not due to a direct effect of the drug on cell growth and that it only occurs in the *in vivo* situation.

The pattern of stimulation of tumour growth by bezafibrate closely follows the serum FFA levels in cachectic animals with maximum stimulation occurring when the animals had lost between 8 and 16% of body weight which coincided with the peak level of serum FFA in these animals (Briddon *et al.*, 1991). However, bezafibrate did not significantly change the plasma FFA levels in tumour-bearing animals. Bezafibrate stimulated lipid accumulation into heart and gastrocnemius muscle in addition to tumour after an oral dose of 1-$^{14}$C-triolein. The increased lipid in the heart most likely arose from an increased accumulation of plasma 18 triglycerol due to an increased tissue level of LPL. The level of LPL in tumour was low and was not increased by bezafibrate. Thus the increased tumour level probably arose from an increased uptake of circulatory FFA as previously suggested (Mermier & Baker, 1974), since fibrate drugs have previously been shown to stimulate uptake of FFA into Ehrlich ascites cells in incubation medium containing albumin (Spector & Soboroff, 1971). The increase in uptake appears to be due to a weakening of the bonds between the FFA and albumin.

The results are consistent with the hypothesis that certain tumours *in vivo* are highly dependent on exogenous lipid for their growth and that mobilisation of host fat stores are an essential factor in this process. If the nature of the lipid product and its role in growth are determined, it may be possible to directly attack the growth of slow-growing solid tumours through this pathway.

This work has been supported by a grant from the Cancer Research Campaign. HDM gratefully acknowledges receipt of a research studentship from the Royal Pharmaceutical Society of Great Britain. We thank Mr M. Wynter for the tumour transplantations.

### References

ABRAHAM, S. & HILLYARD, L.A. (1983). Effect of dietary 18-carbon fatty acids on growth of a transplantable mammary adenocarcinoma in mice. *J. Natl Cancer Inst.*, **71**, 601.

BECK, S.A. & TISDALE, M.J. (1987). Production of lipolytic and proteolytic factors by a murine tumor-producing cachexia in the host. *Cancer Res.*, **47**, 5919.

BIBBY, M.C., DOUBLE, J.A., ALI, S.A., FEARON, K.C.H., BRENNAN, R.A. & TISDALE, M.J. (1987). Charcterisation of a transplantable adenocarcinoma of the mouse colon producing cachexia in recipient animals. *J. Natl Cancer Inst.*, **78**, 539.

BRIDDON, S., BECK, S.A. & TISDALE, M.J. (1991). Changes in activity of lipoprotein lipase, plasma free fatty acids and triglycerides with weight loss in a cachexia model. *Cancer Lett.*, **57**, 49.

FALLON, H.J., ADAMS, L.L. & LAMB, R.G. (1972). A review of studies on the mode of action of clofibrate and betabenzalbutyrate. *Lipids*, **7**, 106.

GLASGOW, W.C. & ELING, T.E. (1990). Epidermal growth factor stimulates linoleic acid metabolism in BALB/c 3T3 fibroblasts. *Mol. Pharmacol.*, **38**, 503.

GREENE, H.L., HERMAN, R.H. & ZAKIM, D. (1970). The effect of clofibrate on rat tissue adenyl cyclase. *Proc. Soc. Exp. Biol. Med.*, **134**, 1035.

LOMBARDI, P., NORATA, G., MAGGI, F.M. & 4 others (1989). Assimilation of LDL by experimental tumours in mice. *Biochim. Biophys. Acta*, **1003**, 301.

LYON, I., KANNAN, R., OOKHTENS, M. & BAKER, N. (1982). Turnover and transport of plasma very-low density lipoprotein triglycerides in mice bearing Ehrlich ascites carcinoma. *Cancer Res.*, **42**, 132.

MANNE, V., ROBERTS, D., TOBIN, A., O'ROURKE, E. & 7 others (1990). Identification and preliminary characterization of protein-cysteine farnesyltransferase. *Proc. Natl Acad. Sci., USA*, **87**, 7541.

MEDES, G., THOMAS, A.J. & WEINHOUSE, S. (1953). Metabolism of neoplastic tissues. IV. A study of lipid synthesis in neoplastic tissue slices *in vitro*. *Cancer Res.*, **13**, 27.

MERMIER, P. & BAKER, N. (1974). Flux of free fatty acids among host tissues, ascites fluid and Ehrlich ascites carcinoma cells. *J. Lipid. Res.*, **15**, 339.

MIDER, G.B., SHERMAN, C.D. Jr & MORTON, J.J. (1949). The effect of Walker carcinoma 256 on the total lipid content of rats. *Cancer Res.*, **9**, 222.

MULLIGAN, H.D. & TISDALE, M.J. (1991). Lipogenesis in tumour and host tissues in mice bearing colonic adenocarcinomas. *Br. J. Cancer*, **63**, 719.

NILSSON-EHLE, P. & SCHOTZ, M.C. (1976). A stable radioactive substrate emulsion for lipoprotein lipase. *J. Lipid Res.*, **17**, 536.

OLLER DO NASCIMENTO, C.M. & WILLIAMSON, D.H. (1986). Evidence for conservation of dietary lipid in the rat during lactation and the immediate period after removal of the litter. *Biochem. J.*, **239**, 233.

ROSE, D.P. & CONNOLLY, J.M. (1990). Effects of fatty acids and inhibitors of eicosanoid synthesis on the growth of a human breast cancer cell line in culture. *Cancer Res.*, **50**, 7139.

SAUER, L.A. & DAUCHY, R.T. (1987*a*). Blood nutrient concentrations and tumor growth *in vivo* in rats: relationship during the onset of an acute fast. *Cancer Res.*, **47**, 1065.

SAUER, L.A. & DAUCHY, R.T. (1987*b*). Stimulation of tumor growth in adult rats *in vivo* during acute strepozotocin-induced diabetes. *Cancer Res.*, **47**, 1756.

SAUER, L.A. & DAUCHY, R.T. (1988). Identification of linoleic and arachidonic acids as the factors in hyperlipemic blood that increase $^{3}$H-thymidine incorporation in hepatoma 7288TC perfused *in situ*. *Cancer Res.*, **48**, 3106.

SPECTOR, A.A. (1975). Fatty acid metabolism in tumors. *Prog. Biochem. Pharmacol.*, **10**, 42.

SPECTOR, A.A. & SOBOROFF, J.M. (1971). Effect of chlorophenoxyisobutyrate on free fatty acid utilization by mammalian cells. *P.S.E.B.M.*, **137**, 945.

STANSBIE, D., BROWNSEY, R.W., CRETTAZ, M. & DENTON, R.M. (1976). Acute effects *in vivo* of anti-insulin serum on rates of fatty acid synthesis and activities of acetyl coenzyme-A carboxylase and pyruvate dehydrogenase in liver and epididymal adipose tissue of fed rats. *Biochem. J.*, **160**, 413.

TISDALE, M.J., BRENNAN, R.A. & FEARON, K.C. (1987). Reduction of weight loss and tumour size in a cachexia model by a high fat diet. *Br. J. Cancer*, **56**, 39.

TISDALE, M.J. & BECK, S.A. (1991). Inhibition of tumour-induced lipolysis *in vitro* and cachexia and tumour growth *in vivo* by eicosapentaenoic acid. *Biochem. Pharmacol.*, **42**, 103.

TSAI, M.-H., YU, C.-L., WEI, F.-S. & STACEY, D.W. (1989). The effect of GTPase activating protein upon ras is inhibited by mitogenically responsive lipids. *Science*, **243**, 522.